# The Effects of Repeated Testing, Simulated Malingering, and Traumatic Brain Injury on High-Precision Measures of Simple Visual Reaction Time

**DOI:** 10.3389/fnhum.2015.00540

**Published:** 2015-11-09

**Authors:** David L. Woods, John M. Wyma, E. William Yund, Timothy J. Herron

**Affiliations:** ^1^Human Cognitive Neurophysiology Laboratory, Veterans Affairs Northern California Health Care SystemMartinez, CA, USA; ^2^UC Davis Department of Neurology, University of California, DavisSacramento, CA, USA; ^3^UC Davis Center for Neurosciences, University of California, DavisDavis, CA, USA; ^4^UC Davis Center for Mind and Brain, University of California, DavisDavis, CA, USA

**Keywords:** aging, motor, head injury, reliability, effort, feigning, computer, timing errors

## Abstract

Simple reaction time (SRT), the latency to respond to a stimulus, has been widely used as a basic measure of processing speed. In the current experiments, we examined clinically-relevant properties of a new SRT test that presents visual stimuli to the left or right hemifield at varying stimulus onset asynchronies (SOAs). Experiment 1 examined test-retest reliability in 48 participants who underwent three test sessions at weekly intervals. In the first test, log-transformed (log-SRT) *z*-scores, corrected for the influence of age and computer-use, were well predicted by regression functions derived from a normative population of 189 control participants. Test-retest reliability of log-SRT *z*-scores was measured with an intraclass correlation coefficient (ICC = 0.83) and equaled or exceeded those of other SRT tests and other widely used tests of processing speed that are administered manually. No significant learning effects were observed across test sessions. Experiment 2 investigated the same participants when instructed to malinger during a fourth testing session: 94% showed abnormal log-SRT *z*-scores, with 83% producing log-SRT *z*-scores exceeding a cutoff of 3.0, a degree of abnormality never seen in full-effort conditions. Thus, a log-SRT *z*-score cutoff of 3.0 had a sensitivity (83%) and specificity (100%) that equaled or exceeded that of existing symptom validity tests. We argue that even expert malingerers, fully informed of the malingering-detection metric, would be unable to successfully feign impairments on the SRT test because of the precise control of SRT latencies that would be required. Experiment 3 investigated 26 patients with traumatic brain injury (TBI) tested more than 1 year post-injury. The 22 patients with mild TBI showed insignificantly faster SRTs than controls, but a small group of four patients with severe TBI showed slowed SRTs. Simple visual reaction time is a reliable measure of processing speed that is sensitive to the effects of malingering and TBI.

## Introduction

Simple reaction time (SRT) tests are basic measures of processing speed that index the minimal time needed to respond to a stimulus (Woods et al., 2015b). SRTs are weakly correlated with general intelligence (Deary et al., 2001) and are slowed in many neurological disorders, including traumatic brain injury (TBI; Stuss et al., 1989b; Willison and Tombaugh, 2006; Neselius et al., 2014), Parkinson’s disease (PD; Camicioli et al., 2008), post-concussion syndrome (Makdissi et al., 2001), cerebrovascular disease (D’Erme et al., 1992), and mild cognitive impairment (Christensen et al., 2005).

In most visual SRT tests, participants respond as rapidly as possible with the keyboard or mouse to stimuli presented at central fixation following randomized stimulus onset asynchronies (SOAs). Here, we describe the clinically-relevant characteristics of a new SRT test (Woods et al., 2015b) that quantifies SRTs separately for stimuli presented in the left and right hemifield, analyzes SRT latencies as a function of the preceding SOAs (Niemi and Naatanen, 1981), and isolates stimulus detection time (SDT) by subtracting the time needed to depress the mouse button (movement initiation time) from the SRT.

The new SRT paradigm was previously used to study age-related (age range 18–82 years) changes in SRTs (Woods et al., 2015b) and revealed shorter SRT latencies (mean = 238 ms) than reported in most prior large-scale studies. In addition, SRT standard deviations (28 ms) were smaller than in previous studies, and within-subject (trial-to-trial) standard deviations were also reduced (53 ms). The differences with previous studies were attributed to the improved precision of the computer hardware and software used for SRT measurement. We also found that SRT latencies increased with age at a rate of 0.55 ms/year, while age effects on SDT latencies were insignificant. SRT latencies decreased by 27 ms as SOAs lengthened, but were minimally affected by the hemifield of stimulation.

The current experiments were designed to evaluate the characteristics of the new SRT paradigm most relevant to its potential clinical deployment: its test-retest reliability, sensitivity to malingering, and sensitivity to the effects of TBI.

### Test-Retest Reliability

In Experiment 1, we examined the test-retest reliability of the new SRT test. Our objective was to compare the test-retest reliability of SRT latency measures with those of previous commercial and non-commercial SRT tests, and to examine the test-retest reliability of the additional measures provided by the new test (e.g., the effects of SOAs on SRT latencies).

### Sensitivity to Malingering

In Experiment 2, we examined the effects of simulated malingering on test performance with the goal of discriminating simulated malingerers from control participants. Based on previous studies that found much longer SRT latencies in malingerers than in either control subjects or brain-injured patients (Kertzman et al., 2006; Willison and Tombaugh, 2006; Reicker, 2008; Marx et al., 2009), we examined the sensitivity and specificity of simple *z*-score cutoffs. We also examined the malingering-detection utility of the additional measures provided by the test.

### The Effects of TBI

In Experiment 3, we evaluated whether the new SRT test would detect impairments in patients with chronic mild and severe traumatic brain injury (mTBI and sTBI). While previous studies of patients in the chronic phase have generally found that SRT slowing is restricted to patients with sTBI (Stuss et al., 1989a; Ferraro, 1996; Bashore and Ridderinkhof, 2002; Tombaugh et al., 2007), we hypothesized that a more sensitive SRT test might also reveal abnormalities in patients with mTBI. We also evaluated whether other performance measures, such as trial-to-trial latency variability, would also show TBI-related abnormalities (Stuss et al., 1989a; Collins and Long, 1996; Tombaugh et al., 2007).

## Experiment 1: Test-Retest Reliability

In Experiment 1, we examined the test-retest reliability of SRT measures in a group of 48 young control participants. Previous studies have generally shown that SRT latencies show high test-retest reliability (Lemay et al., 2004; Sakong et al., 2007). However, little is known about the test-retest reliability of SRT latencies for laterally-presented stimuli, or the test-retest reliability of the additional SRT measures gathered in the current paradigm, including SDTs and the increase in SRT latencies that occurs when stimuli are presented at short SOAs.

Minimal learning effects of SRTs have been found in previous studies (Lemay et al., 2004; Straume-Naesheim et al., 2005; Sakong et al., 2007; Eckner et al., 2011). Here, we examined whether learning would occur in a somewhat more complex SRT paradigm.

### Timing Precision and Replicability

Although the timing calibration of computer hardware is essential for providing accurate estimates of SRT latencies (Plant and Quinlan, 2013), timing calibration data have not previously been published for commercial or non-commercial SRT paradigms. Hardware delays are generally constant for different tests performed on a given computer, but can change substantially (e.g., by 40 ms or more) if the same test is run with a different computer monitor and response device (Plant and Turner, 2009). We therefore provided additional information about hardware and software timing precision for the SRT tests reported here.

Computer software can also introduce timing imprecision when multiple operations (e.g., monitoring for responses, loading files from disk, writing data to disk, etc.) occur concurrently and when paradigm execution is interrupted by other processes executing concurrently on the test computer. Unlike hardware delays, software delays occur unpredictably, and hence need to be measured for each stimulus and response event during a test to assure optimal precision. Previous tests used to evaluate monitor precision have found infrequent software delays of 17–51 ms that vary with SOA and the software platform used (Garaizar et al., 2014). However, software delays have not been measured in more complex paradigms where multiple operations occur concurrently. Here, we provide measures of the software delays associated with each stimulus and response event during SRT test execution.

### Methods

#### Participants

The demographic characteristics of the participants are shown in Table 1. The 48 young volunteers (mean 26.2 years, range 18–46 years, 48% male) were recruited from advertisements in the San Francisco Bay Area on Craigslist (sfbay.craigslist.org), and from pre-existing control populations. All participants were required to meet the following inclusion criteria: (a) fluency in the English language; (b) no current or prior history of psychiatric illness; (c) no current substance abuse; (d) no concurrent history of neurologic disease known to affect cognitive functioning; (e) auditory functioning sufficient to understanding normal conversational speech; and (f) visual acuity normal or corrected to 20/40 or better. They were recruited alongside a larger control population (Experiment 2 in Woods et al., 2015b) and had agreed to participate in four test sessions: three test sessions to evaluate test-retest reliability, and a fourth session to study the effects of malingering (see “Experiment 2”, below). All participants signed written consent forms approved by the institutional review board (IRB) at the Veterans Affairs Northern California Health Care System (VANCHCS), and were compensated for their participation. Fifty eight percent of the participants were college students, and the group as a whole was very well-educated (mean 15.1 years of education). Ethnically, 68% were Caucasian, 11% Hispanic, 9% African American, 9% Asian, and 3% other. The data were compared with those from a normative control group of 189 participants ranging in age from 18 to 82 years whose results have been described in detail elsewhere (Woods et al., 2015b).

**Table 1:** **Demographic characteristics of the normative population (Norm) and the participants in the three experiments**.

Experiment	Group	N	Ages (years)	Education (years)	Male (%)
Norm	Control	189	18–82; 41.0 (21.3)	10–20; 14.6 (2.2)	42%
Experimemts 1 and 2	Control/Malinger	48	18–46; 26.2 (5.6)	12–18; 15.1 (1.9)	48%
Experiment 3	mTBI	22	20–61; 31.0 (11.9)	10–18; 13.4 (1.8)	100%
	sTBI	4	25–57; 39.0 (11.8)	12–16; 14.0 (2.0)	75%

*Normative data refers to subjects in Experiment 2 of Woods et al. (2015b). mTBI, mild TBI; sTBI, severe TBI. Range, mean, and variance are shown for age and education*.

#### Methods and Procedures

**Figure 1:**
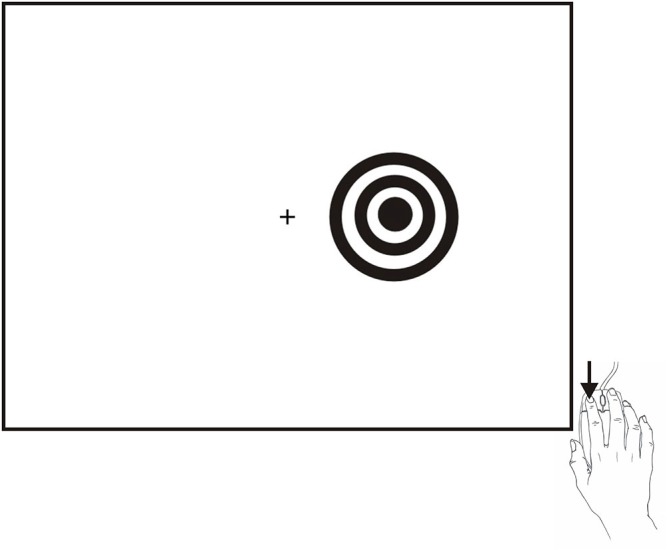
**The SRT paradigm.** Stimuli were high-contrast bulls-eyes presented to the left or right hemifield for a duration of 200 ms at randomized stimulus onset asynchronies (SOAs) ranging from 1000–2000 ms in five 250 ms steps. Stimuli could occur in the visual hemifield ipsilateral (shown) or contralateral to the responding hand.

SRT testing occurred midway through a series of tests that required approximately 2 h to complete^1^. The SRT paradigm is shown in Figure 1 and is available for download at www.ebire.org/hcnlab/cognitive-tests/SRT. Participants responded to the occurrence of a bulls-eye stimulus in either the left or right hemifield by pressing the response button of the mouse with their index finger. After 20 practice trials, 100 stimuli of 200 ms duration were presented randomly to the left and right hemifield at SOAs ranging from 1000 to 2000 ms in 250 ms steps.

A response window of 110–1000 ms was used. Responses outside this range were categorized as false alarms (FAs). The failure to respond during the 110–1000 ms interval following the presentation of a stimulus was categorized as a miss. Hit rate was defined as the percentage of stimuli associated with valid responses. For each participant, hit-rate, false-alarm rate, and mean SRT latency were calculated along with trial-to-trial SRT variance.

While our primary focus was on SRT latency, we were also interested in measuring SDT, the difference between SRTs and movement initiation times measured in a finger-tapping task performed on the same day of testing (Hubel et al., 2013a,b). In addition, we evaluated the reliability of other potentially useful metrics, including hit rate, trial-to-trial SRT standard deviations, Coefficient of Variation (CV, trial to trial standard deviations/mean RT), differences between SRT latencies for stimuli presented to the left and right hemifield, and differences between stimuli presented at short and long SOAs. Other methodological details have been described elsewhere (Woods et al., 2015b).

#### Hardware and Software Calibration

There are two principal sources of hardware delay. First, there is a delay in the appearance of the stimulus after the computer video card sends the stimulus image to the LCD monitor, which depends on monitor electronics. We measured the delay for the 17″ Samsung Syncmaster monitor with a photodiode (StimTracker, Cedrus, San Pedro, CA, USA) and found a mean delay of 11.0 ms (sd = 0.1 ms). Second, there is a variable delay between the moment that the response button is pressed and the moment that the response is registered by the device driver and detected by the computer software controlling the paradigm. The magnitude of this delay (often 20 ms or more) depends on mouse design and the device driver software that signals responses to the operating system (Plant et al., 2003). In the current experiment, we used a PC gaming mouse (Razer Sidewinder, Carlsbad, CA, USA) that required minimal (2.0 mm) movement for button closure and incorporated a device driver with a high USB sampling rate (1.0 kHz). We measured response delays by disassembling the mouse and simulating button closure with an electronic relay. The average response delay was 6.8 ms (sd = 1.8 ms). Thus, total delays introduced by the video display and response device were 17.8 ms.

In addition to hardware delays, stimulus-delivery software can introduce unpredictable delays and latency variability. Presentation software (Neurobehavioral Systems, Inc., Berkeley, CA, USA) is designed so that resource-demanding operations (e.g., loading a stimulus from disk) are multiplexed with continuous high-precision monitoring for event occurrences. This feature enables Presentation to report event times with 0.1 ms precision using the 100 kHz programmable clock. Event-time uncertainties, the difference between times recorded before the event occurred and times recorded after the event, are also recorded for each event. Thus, there will be a gap in the otherwise continuous timing record and a corresponding increase of the event-time uncertainties if stimulus delivery or response monitoring is interrupted by a resource-demanding operation or an extraneous process. For example, if a response occurred during a 5.0 ms interruption, its latency would be logged at the beginning of the interruption and would be associated with a 5.0 ms event-time uncertainty. In the current experiments, the PC was configured to minimize extraneous operating system interruptions. Event-time uncertainties for 5,279 stimulus presentations in Experiment 1 averaged 0.16 ms (sd = 0.05 ms) with a maximal uncertainty of 2.2 ms, and the mean uncertainty for 5,226 response events was 0.22 ms (sd = 0.11 ms) with a maximal uncertainty of 1.3 ms.

#### Data Analysis

The distribution of mean SRTs was asymmetrical (skew = 0.90) so that SRTs were first log-transformed to reduce skew. The examination of the normative data of participants ranging in age from 18 to 82 years (Woods et al., 2015b) showed that both age (*r* = 0.34, *t*_(187)_ = 4.94, *p* < 0.0001) and computer-use (*r* = −0.28, *t*_(187)_ = 3.99, *p* < 0.0001) had significant effects on log-transformed SRT latencies. When analyzed conjointly, these factors accounted for 16% of log-SRT variance (*r* = 0.40) in the normative population, with both age (*t*_(186)_ = 4.26, *p* < 0.0001) and computer-use (*t*_(186)_ = −3.08, *p* < 0.003) independently influencing log-SRTs. Therefore, the regression functions from the normative data were used to calculate log-SRT *z*-scores after correcting for the influence of age and computer-use (see Table 2). SDTs (skew = −0.90) were not significantly influenced by either age (*r* = −0.07) or computer-use (*r* = 0.01), so no regression functions were applied when calculating SDT *z*-scores.

**Table 2:** **Mean values for all experiments**.

Group	Norm	Experiment 1a	Experiment 1b	Experiment 1c	Experiment 2 SM	Experiment 3 mTBI	Experiment 3 sTBI
N	189	48	48	48	48	22	4
Age	41.0	26.2	26.2	26.2	26.2	34.1	46.0
SRT (ms)	237.8	231.5	231.7	228.9	453.2	228.0	280.0
SRT SD (ms)	27.8	17.7	16.7	18.3	123.4	20.9	42.0
Log-SRT z	0.00	0.10	0.11	−0.02	6.25	−0.30	1.39
ISSD (ms)	52.7	46.2	45.1	44.4	172.3	53.6	67.3
CV	21.9%	19.9%	19.4%	19.3%	38.3%	21.6%	28.2%
Accuracy	97.2%	98.4%	98.5%	97.6%	81.8%	96.4%	96.5%
SDT (ms)	138.3	142.1	140.0	130.8	252.2	126.6	171.1
S-L (ms)	26.9	27.9	32.8	34.0	55.6	32.9	58.0

Norm, Normative values from a previous experiment (Experiment 2 in Woods et al., 2015c). Experiments 1a, 1b, and 1c, the three successive test sessions in Experiment 1. Experiment 2, SM, Simulated malingering. Data from Experiment 3 are presented separately for mild and severe TBI patients (mTBI and sTBI). Log-SRT-z, z-score of age- and computer-use regressed log SRT. ISSD, intrasubject (trial-to-trial) standard deviation; CV, coefficient of variation; SDT, stimulus detection time, the difference between SRT and movement initiation time measured in a finger tapping task. S-L, difference in RTs between short (1000 ms) and long (2000 ms) SOAs.

Statistical analysis was performed with multifactor mixed analysis of variance (ANOVA). Separate ANOVAs were performed for different metrics, with Greenhouse-Geisser corrections of degrees of freedom used in computing *p* values in order to correct for covariation within factors or interactions. Effect sizes are reported as partial ω^2^ values or as Cohen’s d. Test-retest correlations were measured with intraclass correlation coefficients (ICCs) using SPSS (IBM, version 22). Pearson product moment correlation coefficients are also reported when appropriate.

### Results

**Figure 2:**
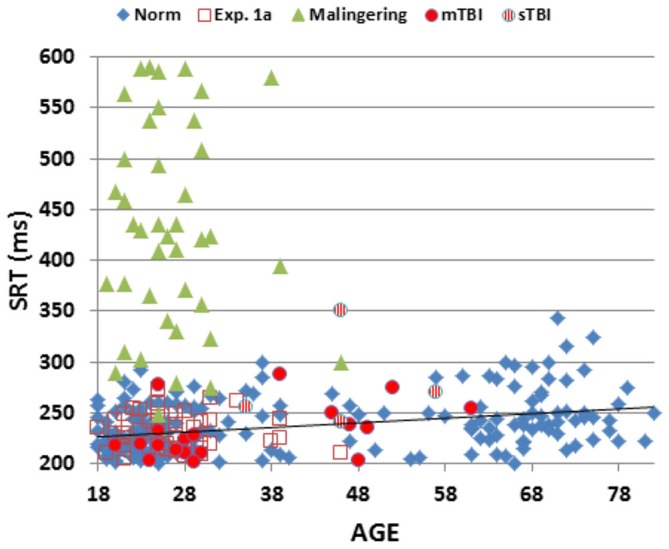
**Mean SRT latencies as a function of age.** SRT latencies from individual participants in normative data (norm, blue diamonds), Experiment 1a (open red squares), Experiment 2 (simulated malingering, green triangles) and Experiment 3 (patients with mTBI, red circles, sTBI, striped red circles). The normative age-regression slope is shown. Simulated malingerers with SRT latencies >600 ms are not included.

**Figure 3:**
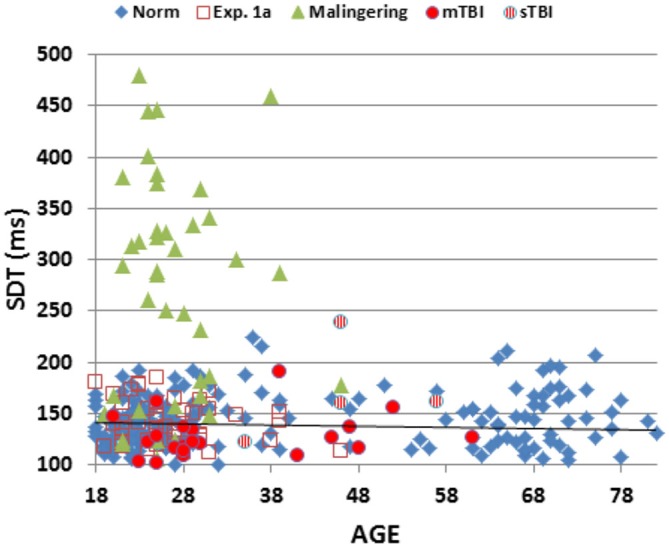
**Mean stimulus detection times (SDTs) as a function of age.** SDTs were derived by subtracting movement initiation time (measured in a finger-tapping experiment performed in the same test session) from SRTs. SDTs are shown for normative data (norm, blue diamonds), Experiment 1a (open red squares), Experiment 2 (simulated malingering, green triangles) and Experiment 3 (patients with mTBI, red circles, sTBI, striped red circles). The normative age-regression slope is shown.

**Figure 4:**
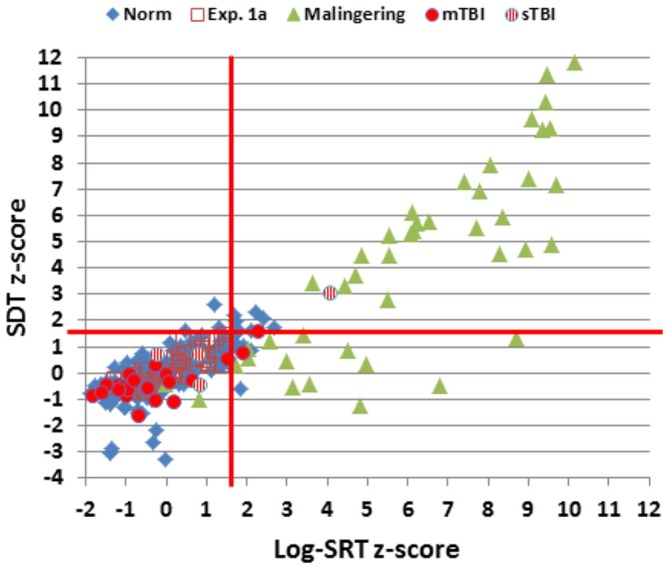
**Log-SRT *z*-scores and SDT *z*-scores for the normative group and the three experiments.** Data from two simulated malingerers with SDT *z*-scores greater than 12.0 and two simulated malingerers with SDT *z*-scores less than −4.0 are not shown. The red lines show *p* < 0.05 thresholds for normative log-SRT and SDT *z*-scores.

Figure 2 shows SRTs as a function of age for the participants in the normative database (blue diamonds) and participants in the first session of Experiment 1 (1a, open red squares). Figure 3 shows SDTs as a function of age, and Figure 4 shows age- and computer-use regressed log-SRT *z*-scores and SDT *z*-scores. Summaries of the results from the different experiments are presented in Table 2.

#### Comparison with Normative Data

We first compared the performance of participants in Experiment 1a (the first test session) with the performance of participants in the normative database (Table 2, norm). No significant group differences were seen in log-SRT *z*-scores (mean *z*-score = 0.10, *F*_(1,234)_ = 1.16, NS), hit rates (*F*_(1,234)_ = 2.46, *p* < 0.15), SDTs (*F*_(1,234)_ = 0.16, NS), or SOA effects (*F*_(1,234)_ = 1.30, NS). However, Experiment 1a participants had slightly reduced CVs (*z*-score = −0.33, *F*_(1,234)_ = 4.92, *p* < 0.03, partial ω^2^ = 0.02) compared to the normative population. In addition, the participants in Experiment 1a were a more homogeneous group than the participants in the normative group, resulting in reduced intersubject standard deviations for SRTs (17.6 ms vs. 27.8 ms) and log-SRT *z*-scores (0.78 vs.1.0). Table 3 shows the percentage of abnormal test results (based on single-sided *p* < 0.05 cutoffs in the normative data). The incidence of abnormal results in Experiment 1a ranged from 0.0–4.2%.

**Table 3:** **Percentage of abnormal results (*p* < 0.05)**.

	L-SRT *z*	Accuracy	CV	S-L	SDT
Experiment 1a	4.2%	4.2%	0.0%	0.0%	0.0%
Experiment 2 (Mal)	93.8%	45.8%	66.7%	43.8%	62.5%
Experiment 3 (mTBI)	13.6%	4.6%	9.1%	4.6%	4.6%
Experiment 3 (sTBI)	25%	25%	0.0%	25%	25%

*Based on p < 0.05 cutoffs established in normative data. See Table 2 for further description of abbreviations*.

#### Test-Retest Reliability

**Figure 5:**
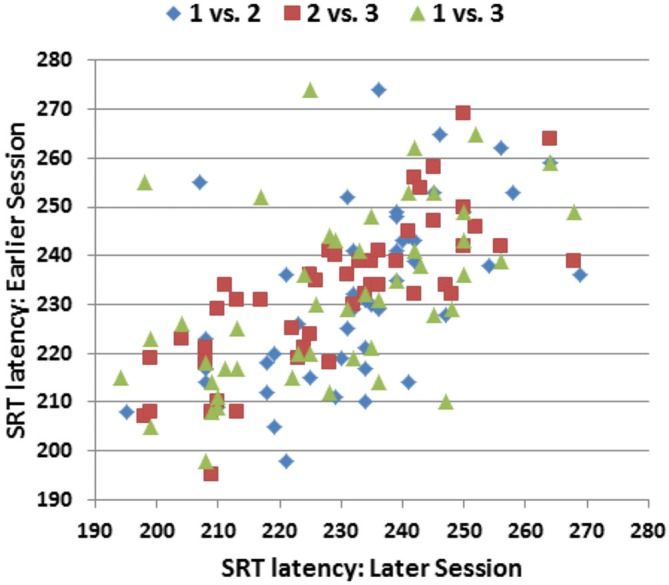
**SRT latencies of individual participants in the three replications of Experiment 1.** The ordinate shows the SRT latencies from the earlier session and the abscissa shows the SRT latencies from the later session. Pearson correlations were *r* = 0.59 (Session 1 vs. Session 2), *r* = 0.80 (Session 2 vs. Session 3), and *r* = 0.53 (Session 1 vs. Session 3).

Figure 5 shows the SRT/SRT plots comparing the performance of individual subjects across the three test sessions. Overall ICCs across the three test sessions were 0.84 for SRT latencies, 0.83 for log-SRT *z*-scores, and 0.87 for SDTs. These high ICCs were consistent with the low within-subject standard deviations across sessions (e.g., 0.33 for log-SRT *z*-scores, a mean within-subject difference of 7.1 ms in SRT latencies across test sessions). Lower test-retest reliability was seen for hit rate (ICC = 0.64), trial-to-trial SRT variance (0.62), CVs (0.67), and the difference in SRT latencies between the longest and shortest SOAs (0.54), while the difference in SRT latencies to stimuli delivered to the left and right visual fields proved unreliable, with an insignificant ICC (−0.10).

#### Learning Effects

Average SRT latencies differed by less than 3 ms across test sessions and there were no significant changes across test sessions for SRT latencies, log-SRT *z*-scores, hit rates, trial-to-trial variance, CVs, or SOA latency differences. However, SDTs shortened slightly from Experiment 1a to Experiment 1c (*t*_(47)_ = 3.86, *p* < 0.0002), due to an unexpected lengthening of movement initiation times (*t*_(47)_ = 4.21, *p* < 0.0001) that occurred in the third session of the companion finger-tapping study (Hubel et al., 2013b).

### Discussion

#### Generalization Across Experiments

Our previous comparison of two large normative populations showed minimal differences in mean SRT latencies (7 ms) that could be accounted for by small differences in paradigm parameters (Woods et al., 2015b). Comparison of the results of Experiment 1 with the normative results from the identical paradigm (Experiment 2 of Woods et al., 2015b) showed no significant differences in log-SRT *z*-scores, hit rates, SDTs, or SOA effects. This suggests that the regression functions developed in the normative population accurately fit the generally younger and better-educated control population tested in Experiment 1.

#### Test-Retest Reliability

The test-retest reliabilities of SRT and SDT measures were similar to those reported in several previous SRT studies. For example, Lemay et al. (2004) found test-retest correlations of 0.80 for three repeated SRT tests, Sakong et al. (2007) found test-retest correlations of 0.78, and Kaminski et al. (2009) found correlations of 0.75 on repeated administration of the SRT test in the Automated Neuropsychological Assessment Metrics (ANAM). The ICCs obtained were also similar to those reported in the CNS Vital Signs reaction time measure (0.80; Gualtieri and Johnson, 2006), but somewhat higher than those reported in CogState (0.65; Eckner et al., 2011) and ImPact (0.57; Resch et al., 2013) tests. The ICCs of SRT *z*-scores also equaled or exceeded the ICCs of manually administered tests of processing speed such as the processing speed test of the NIH Toolbox (Carlozzi et al., 2014) and the WAIS processing speed index (Iverson, 2001).

Consistent with previous reports (Lemay et al., 2004), we found that trial-to-trial variance, CV, and hit rate were less reliable metrics than mean SRT. We also found that the effect of SOA was less reliable than the SRT latency measurements themselves. As expected, differences in SRTs in the left and right visual fields were small and variable in the control participants, and did not correlate significantly across test sessions.

#### Learning Effects

We found no evidence of learning effects on repeated SRT testing, consistent with most previous studies (Lemay et al., 2004; Kida et al., 2005). However, small reductions in SRT latencies have been reported in tests that measure SRTs with fewer trials (Kaminski et al., 2009) or examine performance over a larger number of repeated tests (Eonta et al., 2011).

#### Computer Hardware and Software Factors Influencing SRT Latencies

Timing calibrations revealed that hardware delays added 18 ms to SRT latencies and software delays were minimal. Hardware delays were minimized in the current experiment by using a computer gaming mouse and a relatively fast LCD monitor. Using different monitors and response devices can add 40 ms or more to measured SRT latencies (Plant et al., 2003). This underscores the importance of hardware calibration in obtaining accurate SRT measurements; i.e., hardware factors could increase SRT latencies by considerably more than one standard deviation (28 ms).

In addition to hardware delays, software interruptions can introduce unpredictable delays that increase SRT latencies and latency variability. Presentation software optimizes timing precision and produced a maximal delay of 2.2 ms in Experiment 1. The incidence of software delays has not been investigated in other computerized neuropsychological tests. However, Garaizar et al. (2014) performed monitor calibration studies and found that delays of one to three video frames (17–54 ms) occurred with other behavioral testing software. These delays were thought to be introduced by resource-demanding operations such as data-logging functions, which occur more frequently during actual behavioral testing than during monitor timing calibration. Because the incidence of software timing errors can vary unpredictably with resource-demanding operations and fluctuations in network traffic, event-time uncertainties should be measured for each stimulus and response event to assure optimal timing precision.

## Experiment 2: Simulated Malingering

In Experiment 2, we examined the effects of simulated malingering on SRT test performance with the goal of evaluating the SRT test as a performance-validity metric. Previous studies have suggested that control participants instructed to malinger (Strauss et al., 1994; Wogar et al., 1998; Reicker, 2008) and patients identified as malingering (Kertzman et al., 2006) produce SRT latencies that greatly exceed those observed in control or patient populations. For example, Willison and Tombaugh (2006) found mean SRT latencies of 285 ms in control subjects and 886 ms in simulated malingerers; an SRT latency cut off of 465 ms showed a sensitivity of 80% in detecting simulated malingerers and 100% specificity in distinguishing control participants from malingerers. In addition, 93% of patients with mTBI and 87% of patients with sTBI were correctly categorized into the non-malingering group. Thus, the sensitivity and specificity of Willison and Tombaugh’s simple SRT latency cutoff was superior to that of many performance-validity metrics (Ylioja et al., 2009; Bashem et al., 2014) and symptom-validity tests (Vickery et al., 2001).

### Methods

All participants in Experiment 1 participated in Experiment 2. After the final test session of Experiment 1, these participants were instructed to perform like a patient with mild TBI consequent to a car accident during a fourth test session the following week. The instructions, which were given once for the entire test battery and have been described before (Woods et al., 2015a), were as follows: “Listed below you’ll find some of the symptoms common after minor head injuries. Please study the list below and develop a plan to fake some of the impairments typical of head injury when you take the test. Do your best to make your deficit look realistic. If you make too many obvious mistakes, we’ll know you’re faking! Symptom list: difficulty concentrating for long periods of time, easily distracted by unimportant things, headaches and fatigue (feeling “mentally exhausted”), trouble coming up with the right word, poor memory, difficulty performing complicated tasks, easily tired, repeating things several times without realizing it, slow reaction times, trouble focusing on two things at once.”

### Timing Precision

The hardware used for testing was identical to that used in Experiment 1. Event-time uncertainties for 5,279 stimulus presentations averaged 0.16 ms (sd = 0.04 ms) with a maximal uncertainty of 1.6 ms. Event-time uncertainties for 4,925 responses averaged 0.19 ms (sd = 0.12 ms), with a maximal uncertainty of 1.6 ms.

### Results

Figures 2–4 include the SRTs, SDTs, and log-SRT and SDT *z*-scores from the simulated malingering participants in Experiment 2 (green triangles). The results of Experiment 2 are summarized in Table 2, and Table 3 shows the incidence of Experiment 2 abnormalities. Mean SRT latencies nearly doubled in simulated malingering conditions (mean SRT = 453 ms, log-SRT *z*-score = 6.25, *F*_(1,234)_ = 640.28, *p* < 0.0001, partial ω^2^ = 0.73), with 94% of malingering participants producing SRTs that were abnormally prolonged relative to the upper *z*-score limit (*p* < 0.05) of the normative group. The majority of malingering participants produced very large abnormalities, with 83% producing *z*-scores exceeding 3.0, and 65% producing *z*-scores exceeding 5.0. As a result, a simple *z*-score cutoff of *z* > 3.0 successfully classified 83% of malingering participants and 100% of control participants.

As shown in Table 2, accuracy was also significantly reduced in simulated malingerers (*F*_(1,234)_ = 103.93, *p* < 0.0001, partial ω^2^ = 0.30), and there was a significant correlation between the magnitude of SRT slowing and the magnitude of accuracy reduction (*r* = 0.48, *t*_(46)_ = 3.71, *p* < 0.001). Among malingering participants with abnormal log-SRT *z*-scores, 54% showed accuracy scores in the abnormal (*p* < 0.05) range. The performance of malingering participants was also less consistent than that of control participants, showing greater mean CVs (*z*-score = 2.40, *F*_(1,234)_ = 187.88, *p* < 0.0001, partial ω^2^ = 0.44). Increased CVs correlated strongly with the degree of SRT latency increase (*r* = 0.71, *t*_(46)_ = 6.84, *p* < 0.0001).

In addition, most malingering participants produced greater latency delays in the SRT task than in the finger tapping task, resulting in a substantial increase in the SDT (mean *z*-score = 3.48, *F*_(1,234)_ = 50.27, *p* < 0.0001, partial ω^2^ = 0.17). However, a small percentage (6.5%) of malingering participants showed the opposite inconsistency and produced negative SDTs: i.e., these participants required less time to respond to a stimulus than to merely press the response button during a finger-tapping task.

### Discussion

Virtually all (94%) of the simulated malingerers showed abnormally prolonged SRT latencies, with most showing very large SRT latency increases. As a result, a simple *z*-score cutoff of *z* > 3.0 showed 83% sensitivity in identifying simulated malingerers and 100% specificity in discriminating malingerers from controls. Similar results have been found in previous studies. For example, Strauss et al. (1994) found that simulated malingerers produced SRTs nearly 300% longer than those of controls, and reported that a simple SRT cutoff was able to accurately classify 96% of malingering and 96% of control participants. Both Willison and Tombaugh (2006) and Reicker (2008) found approximately fourfold increases in the SRTs of simulated malingerers and reported that simulated malingerers could be distinguished from controls with high sensitivity and specificity using SRT cutoffs. Similar effects are seen in patients suspected of malingering: Kertzman et al. (2006) found that SRTs were more than twice as long in malingering than non-malingering patients. Moreover, these and other investigators have noted that while neurological patients with MS (Reicker et al., 2007), severe TBI (Ferraro, 1996; Tombaugh et al., 2007), and other neurological disorders (Papapetropoulos et al., 2010) may produce SRTs that are substantially prolonged relative to control participants, their SRT latencies generally remain much lower than those typically seen in simulated malingerers (Willison and Tombaugh, 2006).

#### Comparison of SRTs and other Malingering Detection Metrics

The sensitivity (83%) and specificity (100%) of a log-SRT *z*-score cutoff (*z*-score > 3.0) was superior to the sensitivity and specificity of performance-validity metrics embedded in digit span testing (Ylioja et al., 2009), the Continuous Performance Test (Ord et al., 2010; Erdodi et al., 2014), and the ANAM (Roebuck-Spencer et al., 2013). The sensitivity and specificity of a simple *z*-score cutoff was also greater than that of most symptom-validity tests that are currently in widespread use (Vickery et al., 2001; Jelicic et al., 2011).

#### The Challenge of Expert Malingerers

Performance-validity metrics and symptom-validity tests are generally resistant to the effects of generic test coaching, where participants are warned that they may be given some tests designed to detect malingering (Jelicic et al., 2011). However, little is known about performance-validity test sensitivity when faced with “expert” malingerers, i.e., individuals who have detailed knowledge of the malingering-detection test and the scoring procedures used to identify participants performing with suboptimal effort. Test subjects may acquire such expertise because highly motivated litigants and/or their attorneys may be concerned about the incidence of false positive diagnoses of malingering in performance-validity tests (Berthelson et al., 2013; Larrabee, 2014), and may therefore research test administration and scoring procedures using the internet (Bauer and McCaffrey, 2006), YouTube videos, open-source publications, and descriptions of performance-validity test procedures in textbooks available from online booksellers.

This raises concerns that expert malingerers may be able to avoid detection on existing performance-validity tests. For example, the strategy of an expert malingerer might be to perform with full effort on the Test of Memory Malingering (Tombaugh, 1996) and then perform with reduced effort on other tests. In addition, expert malingerers might titrate their effort on other neuropsychological tests to avoid detection with embedded performance-validity metrics such as reliable digit span (Whitney et al., 2009).

Two features of the SRT test would make it difficult for even expert malingerers to produce abnormal SRT results without detection. First, successful malingering would require precise, conscious control of SRT response latencies. For example, an average participant would need to increase SRT latencies by approximately 45 ms to produce log-SRT *z*-scores in the abnormal range, but would need to avoid increasing SRT latencies by more than 84 ms to assure that *z*-scores remained below the malingering detection cutoff. In other words, the increase in SRT latencies would need to fall within a 40 ms latency window. It is unlikely that even expert malingerers would be capable of such precise SRT latency control, particularly in a paradigm with randomly varying stimulus locations and SOAs, as unconscious (trial-by-trial) SRT latency standard deviations averaged 53 ms in participants performing with full effort.

Second, expert malingerers would need to adjust performance relative to their unknown SRT latencies in full-effort conditions. For example, a participant with short-latency SRTs in full-effort conditions might need to increase SRT latencies by 90 ms or more to produce *z*-scores in the abnormal range, while a participant with long-latency SRTs in full-effort conditions might produce *z*-scores > 3.0 with additional malingering delays of 50 ms or less. Thus, even if it were possible for an expert malingerer to precisely increase SRT latencies by a desired amount, successful malingering would also require that the malingerer possess an accurate estimate of their full-effort SRT latencies. Moreover, malingering participants would need to avoid softer signs of malingering by maintaining high accuracy, minimizing trial-to-trial SRT variance, and producing comparable delays in finger-tapping and SRT studies. In short, malingering on the SRT test without detection would be a very challenging task, even for a fully informed, expert malingerer.

#### Limitations

As in previous studies of simulated malingering (Willison and Tombaugh, 2006), participants were provided with information about the symptoms of TBI which included slowed processing speed, and were warned to make their impairments plausible. However, unlike simulated malingerers in most previous studies, the participants in Experiment 2 were familiar with the SRT test due to repeated test exposure in Experiment 1. This familiarity may have provided them with increased insight about their baseline levels of performance and made it easier for them to concentrate on malingering during Experiment 2.

## Experiment 3: The effects of Traumatic Brain Injury

In Experiment 3, we evaluated the sensitivity of the new SRT paradigm to the long-term effects of TBI. SRT latencies are increased in patients with both mild and severe TBI when tested in the acute phase (Warden et al., 2001; Fong et al., 2009; Bryan and Hernandez, 2012). However, when tested in the chronic phase (more than 6 months post-injury), SRT latency prolongations have been found in patients with severe TBI (sTBI; Stuss et al., 1989a; Ferraro, 1996; Bashore and Ridderinkhof, 2002; Tombaugh et al., 2007), while patients with mild TBI (mTBI) show SRT latencies within the normal range (Incoccia et al., 2004; Willison and Tombaugh, 2006; Tombaugh et al., 2007; Ivins et al., 2009). In addition, previous studies have reported increased trial-to-trial SRT variance in patients with both mild and severe TBI when tested in the chronic phase (Stuss et al., 1989a; Collins and Long, 1996; Tombaugh et al., 2007).

### Methods

#### Participants

Twenty eight Veterans with a history of TBI were recruited from the local patient population. The patients included 27 males and one female between the ages of 20 and 61 years (mean age = 35.2 years) with an average of 13.9 years of education (Table 1). The patients had suffered TBIs of varying severity and etiology, as detailed in Table 4. All participants had suffered head injuries and transient alterations of consciousness, and all were tested more than 1 year post-injury (range 18 months to 24 years). Twenty four of the patients had suffered one or more combat-related incidents with a cumulative loss of consciousness less than 30 min, hospitalization less than 24 h, and no evidence of brain lesions on clinical MRI scans. These patients were categorized as mTBI. The four remaining patients had suffered accidents with hospitalization of one to several months, coma duration exceeding 8 h, post-traumatic amnesia exceeding 72 h, and evidence of brain lesions on MR scans (Turken et al., 2009). These patients were categorized as sTBI. All patients signed written consent forms approved by the IRB at the Veterans Affairs Northern California Health Care System (VANCHCS), and were compensated for their participation. They were informed that the study was for research purposes only and that the results would not be included in their official medical records. Evidence of posttraumatic stress disorder (PTSD), as reflected in elevated scores (>50) on the Posttraumatic Stress Disorder Checklist (PCL), was evident in more than 50% of the TBI sample (Table 4).

**Table 4:** **TBI patient characteristics**.

ID	Age	Edu	Etiology	TBI	PCL	SRT	Hit rate
PAT001^c^	35	12	MVA	Severe	59	256	92%
PAT002^c,d^	24	12	Blast	Mild	54	204	94%
PAT003^c,d^	28	12	Blast	Mild	66	224	94%
PAT005^d^	46	12	MVA	Severe	42	242	100%
PAT012^c,d^	57	16	MVA	Severe	56	271	94%
PAT014	30	14	MVA	Mild	–	211	96%
PAT038^c^	52	18	MVA	Mild	27	275	99%
PAT051^c,d^	41	14	Blast^a^	Mild	45	199	94%
PAT062	20	14	Blast^a^	Mild	41	218	96%
PAT078^b,c^	46	14	MVA	Severe	46	351	100%
PAT081^d^	25	14	Fall	Mild	–	218	80%
PAT101	28	13	Blast	Mild	47	212	98%
PAT106^d^	25	14	Blast	Mild	57	233	98%
PAT109	29	10	Blast	Mild	54	228	95%
PAT110^c,d^	47	14	Blast^a^	Mild	52	239	96%
PAT111	28	12	Fall	Mild	43	211	97%
PAT112^c^	29	14	Blast	Mild	27	202	96%
PAT113^d^	61	16	MVA^a^	Mild	52	255	100%
PAT114^c,d^	27	14	Blast	Mild	72	213	98%
PAT115^c,d^	48	13	Blast	Mild	59	204	100%
PAT117^c^	49	12	Fall	Mild	47	235	100%
PAT120^c^	28	14	Fall	Mild	68	199	97%
PAT122^c,d^	39	16	MVA	Mild	64	288	100%
PAT123^c,d^	25	12	Blast^a^	Mild	72	278	97%
PAT124	45	14	Blast	Mild	60	250	98%
PAT125^c,d^	23	14	Fall	Mild	67	219	99%

*TBI, traumatic brain injury; PCL, TSD Checklist. Age in years. Edu, years of education. MVA, moving vehicle accident.* ^a^*Multiple TBIs;* ^b^*Female;* ^c^*Chronic Pain;* ^d^*Sleep Problems. Hit rate = percent correct*.

Two patients with mTBI produced markedly delayed SRTs (log-SRT *z*-scores of 8.78 and 8.10) suggestive of malingering. These patients had also shown evidence of suboptimal effort on other cognitive tests performed on the same day of testing (Woods et al., 2011, 2015a,c; Hubel et al., 2013b), and their data were excluded from further analysis.

#### Test Procedures

Test procedures were identical to those of the first test session in Experiment 1.

#### Timing Precision

The hardware used for testing was identical to that used in Experiment 1. Event-time uncertainties for 2,999 stimulus presentations averaged 0.13 ms (sd = 0.57 ms). Two stimuli occurred with event-time uncertainties that exceeded 0.4 ms, including one stimulus with a timing uncertainty of 31.5 ms. Event-time uncertainties for 3,015 response events averaged 0.2 ms (sd = 0.7 ms), with four responses showing timing uncertainties in excess of 1.0 ms, and one response with a timing uncertainty of 31.4 ms.

#### Data Analysis

We compared SRT performance in the mTBI and sTBI patient groups with the participants from the normative population and Experiment 1 using ANOVAs.

### Results

SRTs and SDTs from the individual patients are included in Figures 2, 3 (mTBI = red filled circles, sTBI = red cross-hatched circles). Log-SRT and SDT *z*-scores from the patients with mTBI and sTBI are shown in Figure 4; along with the data from the other participant groups. Mean performance measures for mTBI and sTBI patient groups are included in Table 2, and the percentages of abnormal results compared to the normative data are included in Table 3.

As seen in Figure 4 and Table 2, the SRT latencies of patients with mTBI were reduced in comparison with those of the normative population (mean log SRT *z*-score = −0.30, standard error of the mean = 0.24), although these differences failed to reach statistical significance either in comparison with the normative group (*F*_(1,209)_ = 1.79, NS) or with the participants in Experiment 1a (*F*_(1,68)_ = 3.04, *p* < 0.09). SDTs showed a similar pattern, with reduced SDTs in mTBI patients that failed to reach significance in comparison with normative controls (*F*_(1,209)_ = 2.61, *p* < 0.11) or the participants in Experiment 1a (*F*_(1,68)_ = 3.31, *p* < 0.08).

In contrast, the small group of four patients with sTBI produced log-SRT *z*-scores that were delayed with respect to the normative population (mean log-SRT *z*-score = 1.39, *F*_(1,191)_ = 7.29, *p* < 0.01, Cohen’s *d* = 0.93), and with respect to the data from control participants of Experiment 1a (*F*_(1,50)_ = 7.90, *p* < 0.01, ω^2^ = 0.12). Moreover, log-SRT *z*-scores in the patients with sTBI were significantly slowed compared to those of patients with mTBI (*F*_(1,24)_ = 6.45, *p* < 0.02, partial ω^2^ = 0.18). There was also a trend toward increased SDTs in patients with sTBI when compared to normative controls (*F*_(1,191)_ = 3.77, *p* < 0.06), and a significant difference between the patients with sTBI and the participants in Experiment 1a (*F*_(1,50)_ = 8.87, *p* < 0.01, ω^2^ = 0.20) and the patients with mTBI (*F*_(1,24)_ = 8.86, *p* < 0.01, partial ω^2^ = 0.24).

Further analysis showed that three patients produced log-SRT *z*-scores in the abnormal (*p* < 0.05) range (Figure 4), including one sTBI patient who produced a log-SRT *z*-score of 4.10, i.e., above the malingering-detection cutoff established in Experiment 2. Three observations suggest that this patient was not malingering: (1) this patient’s accuracy was superior to that of any of the simulated malingerers in Experiment 2; (2) the patient’s CV was reduced below mean control levels (*z*-score = −1.12) and below those seen in simulated malingerers; and (3) this patient did not show evidence of malingering on other cognitive tests (Woods et al., 2011, 2015a; Hubel et al., 2013b). SRT elevations of similar magnitude have been noted in patients with sTBI in previous studies (van Zomeren and Deelman, 1976; Willison and Tombaugh, 2006).

Trial-to-trial variations in SRT latency, reflected in the CV, did not differ between the normative control group and patients with either sTBI or mTBI, but CVs were increased in both TBI groups relative to the CVs of participants in Experiment 1a (for sTBI, *F*_(1,50)_ = 6.54, *p* < 0.02, partial ω^2^ = 0.10; and for mTBI *F*_(1,68)_ = 8.66, *p* < 0.005, partial ω^2^ = 0.10).

### Discussion

Previous studies have shown that the SRTs of patients with mTBI generally fall within the normal range. We found that SRTs of patients with mTBI tended, if anything, to be slightly faster than those of control populations. One possible explanation is the patients in our mTBI group were military veterans, and most had been deployed in combat. Previous studies have suggested that SRTs may be shortened by military deployment (Vasterling et al., 2006) and combat exposure (Marx et al., 2009).

SRTs of one patient with sTBI showed significant slowing, consistent with previous studies (van Zomeren and Deelman, 1976; Willison and Tombaugh, 2006), and two other patients with sTBI showed SRTs in the upper normal range. These results are also consistent with the recent meta-analysis of Puopolo et al. (2013), who found evidence of systematic delays in a sensory-motor component of reaction time studies in patients with sTBI.

We found equivocal increases in SRT CVs when the data from both mTBI and sTBI groups were compared to the results of Experiment 1a, but no significant differences with respect to the normative control group. One explanation for these equivocal findings is that CVs increased with age in the normative population (*r* = 0.27, *t*_(187)_ = 3.84, *p* < 0.0001), so that the inclusion of older participants in the normative population increased the mean normative CV. Increased trial-to-trial variability has previously been reported in patients with TBI who otherwise show normal performance on neuropsychological tests (Collins and Long, 1996), and longitudinal studies have found that increased SRT variability may persist for up to 10 years after head injury (Hetherington et al., 1996). However, the test-retest reliability of the CV was relatively low (see “Experiment 1” Section), suggesting that CV increases in individual patients would have relatively limited diagnostic use.

#### Malingering Detection in Studies of TBI Populations

We excluded the results of two patients with TBI based on evidence of malingering on other tests and signs of suboptimal effort on the SRT test. Both patients had volunteered for research studies and had been told that their results would be confidential and not used for clinical purposes. These results highlight the importance of incorporating performance-validity and/or symptom-validity measures in TBI studies. The inclusion of these two participants would have resulted in a significant increase in the mean log-SRT *z*-scores of the mTBI group.

#### Limitations

Due to the small sample size and modest effect sizes, the current findings should be considered tentative. In addition, the majority of the veteran patients with TBI had evidence of clinically significant PTSD symptoms (e.g., PCL scores > 50 see Table 4), which may have influenced performance (Kertzman et al., 2014; Verfaellie et al., 2014) and increased performance variability (Swick et al., 2013). In addition, many of the patients had sleep disturbances which can also impair performance on speeded response tasks (Waters and Bucks, 2011).

## Discussion

### A Comparison of SRT Tests

The computerized SRT test described here has a number of desirable features that distinguish it from other SRT tests. First, it produced SRT latencies that were shorter than those obtained with other SRT tests (Woods et al., 2015b). Second, it showed good replication across large normative populations (Woods et al., 2015b) and between the participants in Experiment 1 and the normative controls. Moreover, test-retest standard deviations of SRT measures were low, and test-retest ICCs generally exceeded those of other SRT tests and manually administered neuropsychological tests of processing speed. Finally, unlike most other SRT tests, the current test enables comparisons of SRT latencies to stimuli presented in the left and right hemifield (e.g., for use in participants with callosal or unilateral lesions), and permits an analysis of performance as a function of the preceding SOA. When coupled with a finger tapping test (Hubel et al., 2013a), it also provides information about SDT.

### The Precision of SRT Latency Measurements

Hardware and software delays were found to contribute 18 ms to measured SRT latencies. Since the SRT latencies that we analyzed showed low standard deviations (18 ms in Experiment 1a participants and 28 ms in the normative control group), the calibration of hardware delay is essential to enable valid comparisons of the normative data with the results obtained using other hardware configurations with the same paradigm.

The influence of software delays has been less thoroughly studied. Garaizar et al. (2014) noted the occurrence of occasional delays of one or two video refreshes (i.e., 17 or 34 ms) when E-Prime and PsychoPy were programmed to present video stimuli at very rapid rates. However, Garaizar’s tests did not analyze delays that may occur in more complex experiments, where multiple program operations (e.g., response polling, randomization, displaying multiple images, storing results, etc.) occur concurrently. Although software delays are unlikely to have a major influence on SRT latency measures in most circumstances, the SRT test that we used provides event-time uncertainty measures for each stimulus and response event and so permits the evaluation of software-timing precision in each test performed.

### Malingering Sensitivity of SRT Testing

Our results confirmed those of previous studies demonstrating that simulated malingerers and patients thought to be malingering produce SRTs with latencies well outside the range of those of control subjects or brain-injured patients (see “Experiment 2 Discussion” Section). In addition, we found that most simulated malingerers showed three additional softer signs of malingering: (1) greater slowing of SRTs than movement initiation times resulting in increased SDTs; (2) reductions in hit rate; and (3) disproportionate increases in trial-to-trial SRT variance. These findings lend support to the argument that SRTs can serve as a useful metric in malingering detection (Willison and Tombaugh, 2006). Indeed, our results suggest that a log-SRT *z*-score cutoff of 3.0 showed a sensitivity and specificity of malingering detection that was superior to that of current performance-validity and symptom-validity tests, and that is also likely to be more resistant to expert malingering (see “Experiment 2 Discussion” Section).

### Traumatic Brain Injury and SRTs

As in previous studies (see “Experiment 3 Discussion” Section), we found that the SRT latencies of patients with mTBI were not significantly different from those of control subjects, while some patients with sTBI produced significant SRT abnormalities. Further studies with larger TBI patient populations are needed to more fully characterize the sensitivity of the new SRT test to abnormalities that may be present in patients with varying severities of TBI.

## Conclusion

We describe a new visual SRT test that presents stimuli to the left and right visual fields at varying SOAs, quantifies SRTs with high precision, permits an examination of the effects of SOA and hemifield of stimulus delivery, and enables the examination of SDT. Three experiments evaluated test-retest reliability, malingering effects, and sensitivity to TBI. The results indicate that the new SRT test provides highly reliable measures of processing speed, can accurately discriminate simulated malingerers from control participants, and reveals normal SRTs in patients with mild TBI, but shows delayed SRTs in some patients with severe TBI.

## Conflict of Interest Statement

DLW is affiliated with NeuroBehavioral Systems, Inc., the developers of Presentation software that was used to create these experiments. The other authors declare that the research was conducted in the absence of any commercial or financial relationships that could be construed as a potential conflict of interest.

## Footnotes

^1^Each test session included the following computerized tests and questionnaires: finger tapping, simple reaction time, Stroop, digit span forward and backward, phonemic and semantic verbal fluency, verbal list learning, spatial span, trail making, vocabulary, design fluency, the Wechsler Test of Adult Reading (WTAR), visual feature conjunction, risk and loss avoidance, delay discounting, the Paced Auditory Serial Addition Task (PASAT), the Cognitive Failures Questionnaire (CFQ), the Posttraumatic Stress Disorder Checklist (PCL), and a traumatic brain injury (TBI) questionnaire.
